# Articaine versus lidocaine inferior alveolar nerve block in posterior mandible implant surgeries: a randomized controlled trial

**DOI:** 10.4317/medoral.25475

**Published:** 2023-02-18

**Authors:** Yakup Gülnahar, Aysan Lektemur Alpan, Evrem Gülnahar

**Affiliations:** 1 OrcID 0000-0001-6583-088X. PhD DDS. Department of Maxillofacial Surgery, Faculty of Dentistry, Erzincan Binali Yıldırım University, Erzincan, Turkey; 2 OrcID 0000-0002-5939-4783. PhD DDS. Department of Periodontology, Faculty of Dentistry, Pamukkale University, Denizli, Turkey; 3 OrcID 0000-0002-9778-8134. PhD DDS. Department of Prosthodontics, Faculty of Dentistry, Erzincan Binali Yıldırım University, Erzincan, Turkey

## Abstract

**Background:**

The aim of this study is to compare the effects of %4 articaine and %2 lidocaine on inferior alveolar nerve block (IANB) for implant surgery in the posterior mandible.

**Material and Methods:**

The patients who have inserted implants in the posterior mandible were divided into 2 groups for IANB: lidocaine and articaine. VAS = visual analog scale, pain during surgery and injection, lip numbness time, mandibular canal-implant apex distance, age, gender, bone density, implant number, release incision, adjacent teeth, and duration of surgery were analyzed using t-test, Mann-Whitney U test, Spearman's coefficient, and, Pearson's chi-squared test. This trial followed the recommendations of the Consort Statement for reporting randomized controlled trials.

**Results:**

577 patients were included and 1185 dental implants were analyzed. There was no significant difference between the two groups in terms of injection and surgery VAS values (*p*>0.05). The lip numbness time of lidocaine was 3.06±3.22min while articaine was found to be 2.96±3.09min (*p*>0.05). Mandibular canal-implant apex distance was found to be 2.28±0.75mm in the articaine and 2.45±0.86mm in the lidocaine group (*p*<0.05). Release incision was made more in the articaine group (51/252) than in the lidocaine group (40/325) (*p*<0.05).

**Conclusions:**

There was no difference between the %4 articaine and %2 lidocaine in terms of pain perception in posterior mandible implant applications. Both anesthetics provided adequate anesthesia for implant application.

** Key words:**Articaine, dental implant, lidocaine, local anesthesia, mandibular nerve, pain perception.

## Introduction

Local anesthesia (LA) is of great significance for modern medicine and dentistry and is applied so often (1). A local anesthetic injection numbs a target area and prevents the patient from feeling pain. Although pain can be successfully managed during dental processes, it is also a common concern among patients. Inferior alveolar nerve block (IANB) is the most used anesthetic method for various dental procedures in the mandibular area. When applied successfully, IANB provides sufficient anesthesia over a large part of the posterior mandible for performing surgical and restorative dental procedures (2). Within the literature, there is no consensus about whether IANB or infiltration anesthesia (INF) should be the preferred method to anesthetize the posterior mandible. However, stress and a pain-free environment are essential for maintaining a positive patient attitude and optimal operator performance in implant surgery. Regarding implant surgery, in comparison with INF, better anesthetic efficiency of IANB has been reported recently (3). Since 1949 lidocaine has been regarded as the golden standard of local anesthetic agents (4). However, thanks to the efforts to develop faster and stronger anesthetics with a shorter halving time, some other alternatives were introduced. Articaine is a common local anesthetic agent used in dentistry (2). Lidocaine is an amide compound that is based on the structure of a benzene ring (C6H6). On the other hand, articaine has a thiophene ring and provides greater lipid solubility and increased efficiency as a larger part of the injected dose can enter target neurons (5). The lipid solubility of articaine can be four times higher than that of lidocaine (1). Though some evidence supports this hypothesis, there is not any consensus on the superior anesthetic efficiency of articaine. Haas *et al* (6) stated that the anesthetic efficacy of articaine is similar to the efficiency of other anesthetics, and it is not superior to the others in both the maxilla and mandible. Nydegger *et al* (7) stated that although articaine is significantly more efficient than lidocaine and prilocaine in INF of mandibular first molars, IANB cannot be considered as an alternative. Maruthingal *et al* (8) showed that although articaine is more effective than lidocaine in pulp and lip anesthesia, it is not significantly better in anesthesia of the lingual tissue of the mandible. Another study showed that anesthesia was achieved in 7.4 minutes with articaine while it took 8.7 minutes for lidocaine (1). It was also claimed that thanks to its protein binding properties, articaine provides a longer duration of anesthesia (9,10). A recent study which was carried out over healthy volunteers by Robertson *et al* shows that, in achieving pulp anesthesia in a mandibular molar, 1:100.000 adrenaline + 4% articaine in buccal infiltration anesthesia is more effective than 1:100.000 adrenaline + 2% lidocaine (11). Based on ongoing controversial studies and the literature mentioned above, the purpose of our study is to evaluate the pain and satisfaction level of implant surgery patients upon IANB application with two different anesthetic agents.

Material and Method

- Study Pattern

The population of the study consists of all patients, who were planned to be applied implants in the zone distal to the mental foramen, between February 4, 2019, and March 7, 2022, at each of the two participating clinics. Two surgeons following the same surgical protocol at two different university hospital with similar settings and properties participated to the study. Procedures were explained to the participants and all of them signed consent forms prior to participating in the study. The study was approved by Pamukkale University Ethical Committee (E-60116787-020-201307). All procedures performed in this study comply with the ethical standards of the institutional and/or national research committees and the 2013 Declaration of Helsinki and its subsequent amendments or comparable ethical standards. A normal healthy patients and patients with mild systemic disease were included the study. Patients who have any hormonal condition, who use any medication, who need further surgery that can change the perception of pain, and who will be sedated because they cannot indicate pain perception, were excluded from the study.

Inclusion Criteria:

1) Patients with systematic conditions are classified as ASA Class I and Class II (12).

2) The patients who need one implant at least due to posterior mandibular tooth loss at least 2mm distal to the mental foramen.

Exclusion Criteria:

1) Pregnancy, lactation, and taking contraceptive pills.

2) Oversensitivity or anaphylactic reactions which contraindicate the intervention.

3) Orofacial neurological symptoms.

4) Infections at the operation zone.

5) Psychotropic medicine, sedative, or NSAI use which can alter the sense of pain.

6) Pathological mental conditions (dementia, psychosis) and lack of cooperation.

7) Advanced surgical necessities (GBR, split crest, vertical regeneration, block graft, etc.)

8) Operations carried out with conscious sedation.

9) Patients who do not want to sign consent.

**Figure 1:**
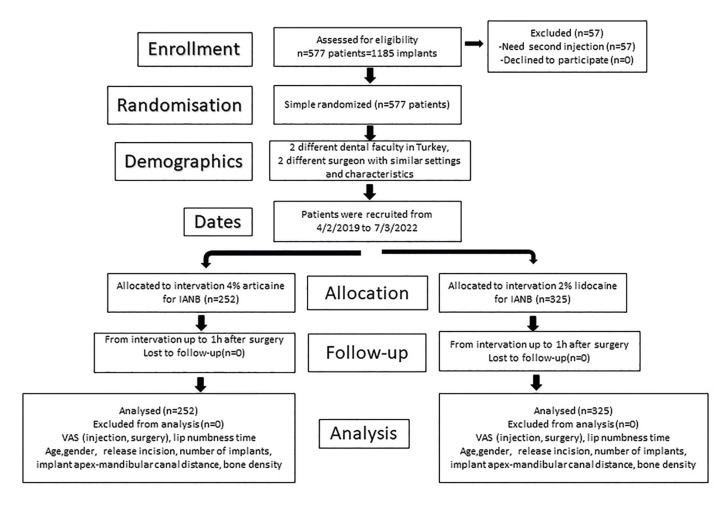
Flow diagram generated in accordance with CONSORT 2010 guidelines.

Participants were randomly assigned to one of the 2 treatment groups (lidocaine and articaine) following a simple software-generated random number procedure via the “List Randomizer” application (https://www.random.org.lists). The primary outcome of the study was to measure the patients’ perception of pain during anesthesia administration and surgery, using a numerical rating scale (VAS) from 0 (no pain) to 10 (the worst pain imagine) (13,14). Potential data which could alter the perception of pain such as gender, age, achieving lip numbness time, use of release incision, number of placed implants, implant apex to the mandibular canal distance, and bone density were recorded (Fig. 1).

- Interventions

The first group was administered 40mg/ml articaine hydrochloride and 0.006mg/ml epinephrine hydrochloride (Ultracain D-S, Sanofi, Paris, France), and the second group was applied 20mg/ml lidocaine hydrochloride and 0.0125 mg/ml epinephrine based anesthesia (Jetokain, Adeka, Turkey). In both groups, operations took less than 60 minutes after the anesthesia. Procedures that lasted longer than 60 minutes and procedures that required advanced surgical applications for implant surgery were excluded from the study. Periosteal integrity was tried to be maintained but the patient’s periosteal integrity which could not be maintained was noted. IANB was applied via a 2 ml capacity 27-G 50 mm standard dental syringe. The patient’s mouth was opened as much as possible so that the raphe pterygomandibularis between the musculus buccinator and musculus constrictor pharynges superior could be seen clearly. From the front edge of the ramus mandibularis, the overlying soft tissue was removed via a mouth mirror. The needle was pushed to the slightly lateral part of the middle zone of the pterygomandibular raphe and contacted the bone.

Then it was slightly pulled and aspirated, and the anesthetic agent was injected. To the participants of both groups, all of the anesthetic agents in the ampoule were injected. Following the injection, if numbness could not be achieved in the lips, a second injection was applied. No extra INF was applied to the buccal zone. Upon achieving adequate numbness, a standard implant placing procedure was followed. With the purpose of exposing the bone, the mucoperiosteal flap was elevated and the zone in which the implant would be placed was drilled at 800-1200 rpm and cooled with saline in accordance with the producer company’s guidelines. Then the implants were screwed to the bone with a pressure of 35nm of torque. Following this step, flaps were primarily closed via 4/0 non-absorbable monofilament polypropylene sutures (Monoprolen, Boz, Ankara, Turkey).

- Data Analysis

With the purpose of reaching reliability power and confidence levels of 95% and to be able to calculate the pain strength at around d = 0.50, the size of the sample was thought to be 184 patients at least 92 patients per group (11). Shapiro-Wilk test was utilized to see whether the variables of groups had a normal distribution. In order to be able to calculate the definitive statistics for all variables of the study, the data was converted into Tables, and, for the continuous variables of the study, mean, SD, minimum, maximum, and median were calculated. Furthermore, absolute frequency and percentages were presented for the categorical ones and analyzed using Pearson's chi-squared test. An independent sample t-test was used to compare the data which was obtained by measurements (lip numbness time, implant-nerve distance), while the Mann-Whitney U test (VAS) was utilized to compare the data which was obtained by scoring. Spearman coefficient was used with the purpose of measuring the correlation between primary and secondary outcomes. The significance level was calculated as *p*<0.05 for all tests.

## Results

**Figure 2:**
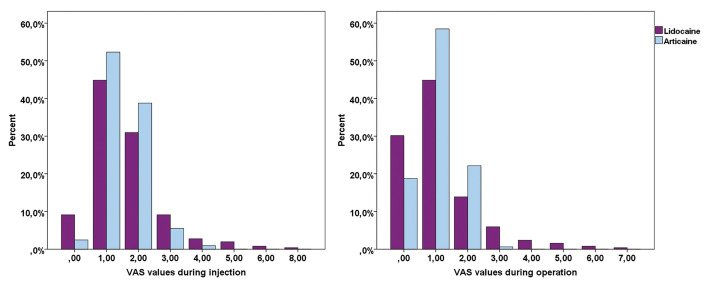
VAS values during injection and operation.

634 patients participated in the study. Who needed a second anesthetic injection, were excluded from the study (57 patients). While 25 (9%) patients needed a second injection in the articaine group, the number of patients who needed a second injection in the lidocaine group was 32 (9%). 577 participants were included in the study and these participants have applied for 1185 dental implants. 43.7% (252 patients) of the participants were in the articaine group while the lidocaine group consisted of 56.3% (325 patients) of the patients (Table 1). The average age of the patients was 45.25±14.99 in the articaine group and 46.43±16.06 in the lidocaine group. In the articaine group, 41,7% (105/252 patients) of the participants were males and 58,3% (147/252 patients) were females (Table 1). For the lidocaine group, the ratio of male and female participants was 63,7% (118/325 patients) and 36,2% (207/325 patients) respectively. While 2.19±0.74 implants per participant were applied in the articaine group, the number was 2.4±0.77 for the lidocaine group. The ratio of releasing incisions was 20,3% for the articaine group, and the releasing incision was performed for 12,3% of the participants in the lidocaine group (Table 1). While 51.2% (129 patients) of the articaine applied zones were on the right mandible, 48,8% (123 patients) were on the left mandible (Table 1). On the other hand, while 44.6% (145 patients) of the lidocaine applied zones were on the right mandible, 55.4% (180 patients) were on the left mandible. 10,7% (27 patients) of the patients who applied articaine had D1 bone type, 84,9% (214 patients) had D2 bone type and 4,4% (11 patients) had D3 bone type. 8,6% (28 patients) of the patients who were administered lidocaine had D1 bone type, 88% (286 patients) had D2 bone type and 3,4% (11 patients) had D3 bone type (Table 1). With the patients who applied IANB, no meaningful difference between VAS scores of articaine (0-4, 1) and lidocaine (0-8, 1) was determined in terms of the felt pain during the application of anesthesia (*p*=0.778) (Fig. 2). Besides, in terms of the felt pain during surgery, there was no meaningful difference among the patients in the articaine (0-3, 1) and lidocaine (0-7, 1) groups (*p*=0.302) (Table 2, Fig. 2). While the average elapsed time for achieving lip numbness with lidocaine was 3.06±3.22 minutes, the average elapsed time for articaine was 2.96±3.09. There was no meaningful difference between the elapsed time for these two anesthetics (*p*=0.678). When the proximity of the mandibular canal was measured, the distance was found to be 2.28±0.75mm in the articaine group and 2.45±0.86mm in the lidocaine group (Table 2). Although the proximity to the mandibular canal was statistically higher in the articaine applied group, this result did not make a difference in the pain perception of the patients. No correlation was determined between proximity to the mandibular canal and patients’ perception of pain.

**Table 1:**
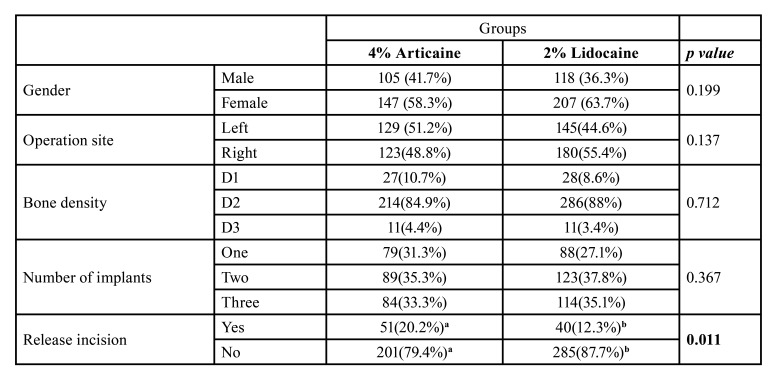
Demographic Profile of the Patient and Characteristics of the Intervention According to the Type of Anesthesia: n (%).

**Table 2:**
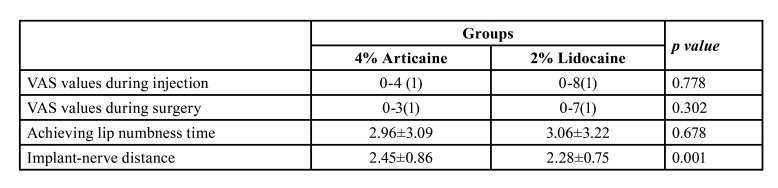
Degree of pain, lip numbness time, implant- nerve distance between the intervention: Median , mean±SD values.

When the correlation of the variables was analyzed, a positive correlation was found between those who felt pain during anesthesia and those who felt pain during surgery in the lidocaine group (r=0.499, *p*=0.001). Furthermore, a positive correlation was discovered between the increase in the duration of anesthesia and the pain felt during surgery (r=0,139, *p*=0.028). In the articaine group, a positive correlation was determined between those who felt pain during anesthesia and those who felt pain during operation (r=0.353, *p*=0.001).

## Discussion

This study, it was aimed to compare the pain level perceived by the patients during implant surgery with patients who applied two different anesthetic agents (lidocaine and articaine) to obtain IANB on the posterior mandible. According to the results of the study, both during the administration of anesthesia and during the operation with patients who underwent IANB, there was no significant difference in terms of pain perception between the two groups. Although the distance to the mandibular canal was significantly reduced in the articaine group and significantly more releasing incisions were applied to these patients, they did not differentiate patients' perception of pain between the two groups. Besides, the amount of the felt pain did not differ in accordance with the bone type. Both anesthetic agents which were used in IANB that were applied for implant placement in the posterior mandible were equally effective. Considering the factors which can affect the perception of pain, there was not any significant difference between the groups in terms of age, gender, and the number of placed implants. The factors affecting the duration of anesthesia are intrinsic properties of the anesthetic agent and method and are directly affected by the pKa value – a smaller pKa value is associated with a shorter delay. pKa value of 4% articaine solution is 7.8 (15). The value for lidocaine is 7.9 (15). However, it is controversial if this difference is the advantage of articaine (16). Within other studies, lapsed time for achieving numbness with articaine is explained as follows: Gregorio *et al* (17), 1.66 minutes, Colombini *et al* (18), 14.29 seconds, and Moore *et al* (19), 2.8 minutes. The recorded numbness time for lidocaine is as follows: Dugal *et al* (20), 1.15 minutes, Dionne (21) 2-3 minutes. In our study, while numbness was achieved in 3.06 minutes on average with lidocaine, the lapsed time for articaine was 2,96 minutes. With different studies, the time for achieving numbness can be measured in different ways. In our study, the time is measured from the second the injection was applied to the tissue.

When the studies comparing the efficiency of the anesthetic agents are evaluated, it can be seen that there is no consensus in the literature. Since implant surgery is performed with sensitive measurements, IANB anesthesia was preferred in our study as the patient’s pain can affect the surgeon’s performance. In a meta-analysis, it was reported that a 2% lidocaine injection in third molar surgery provided a higher VAS score than a 4% articaine injection (22). In a study comparing the effect of articaine and lidocaine applied with buccal infiltration anesthesia to irreversible pulpitis patients, the use of 4% articaine as both IANB and buccal infiltration, 2% lidocaine as IANB with buccal infiltration (50%) or IANB alone recorded the highest success rate (70%) when compared to 2% lidocaine (30%) (23). In cases where the relevant tooth needs to be anesthetized in both tooth extraction and endodontic treatments, structures that are different from implant surgery come into question. In irreversible pulpitis, IANB failure may be experienced due to local acidosis or activation of nociceptors because of inflammation. There are studies stating that IANB may not be necessary for standard implant surgery in the posterior mandible, and infiltration anesthesia with 4% articaine 1:100.00 epinephrine may be sufficient (24). In their study, Garcia-Blanko *et al* (3) compared the effects of 4% articaine during both infiltrative anesthesia and mandibular anesthesia. The mean VAS values felt by the patients were 0.4±0.8 in the mandibular anesthesia group and 0.7±1.1 in the infiltration group, and statistically, the patients felt more pain in infiltration anesthesia (3). In the same study, it was found that the distance from the mandibular canal was unrelated to the pain felt, in line with our study. The authors have recommended the application of IANB if deeper anesthesia is desired in the posterior mandible. Malamed *et al* (25) compared the effects of articaine and lidocaine injections in pediatric patients. VAS scale data for both simple and complicated procedures did not provide statistical significance between groups. Rebolledo *et al* (26) performed a split-mouth study with the purpose of determining the efficiency of 2% lidocaine and 4% articaine anesthesia during surgical removal of an impacted mandibular third molar in terms of IANB. This study was conducted on 30 patients with symmetrical impacted mandibular wisdom teeth. Despite the positive effects of 4% articaine, no significant difference was found between the anesthetics in terms of intraoperative pain, the onset of anesthesia and the volume of the anesthetic solution used, and the need for re-anesthesia. In our study, similar to the study above, no difference was determined in terms of perceived pain. Vishal *et al* (27) tested two anesthetic agents on 100 patients during mandibular molar extraction. Numbness was achieved with articaine at an average of 3.24±0.45 while the lapsed time for achieving numbness with lidocaine was 3.65±0.39. In comparison with the lidocaine group, less pain was felt by the patients in the articaine group (*p*<0.05). However, groups did not show any differences in terms of pain perception after 5, 15, 30, and 60 minutes following the anesthesia. In our study, during and after the anesthesia, there was not any difference between the groups. This may stem from the difference in the applied IANB method or the dentist applying it. During the anesthesia, which is applied so close to the bone, the possibility of feeling pain may increase. In our study, it was found that the patients who felt pain during anesthesia also felt pain during the operation. In line with ethical frameworks, the operation cannot be continued even though patients feel pain. Pain perception can vary from person to person. Some people are more sensitive to pain, so they focus on the pain and feel it more as a consequence. Because IAN is both a motor and a sensory nerve by nature, it is essential in enabling people to interact with the environment because they provide sensations of touch, pressure, temperature change, and pain (3). Patients may also interpret the vibrations and pressure felt during the operation differently, which is the limitation of the study. Fatigue, anxiety, and pain are interrelated. Increased levels of fatigue and anxiety are also responsible for increased pain perception (28). Therefore, the anxiety level of the patient, who knows that he/she will have an operation, may have changed his/her perception of pain. The thiophene ring structure of articaine has been hypothesized to provide superior and unique abilities to penetrate bone and other tissues (29). Meechan showed that, although articaine has the ability to penetrate through the bone cortex and induce anesthesia in the lingual tissue, bilateral buccal and lingual injections are more effective.

Epinephrine is commonly added to local anesthetic solutions to induce vasoconstriction at the injection site. Addition of epinephrine increases local anesthetic activity by antagonizing the natural vasodilator effects of most local anesthetics, in addition to the vasoconstrictive effects provided by α-1 adrenoceptors. Moore *et al* (19) investigated the anesthetic effect of articaine, 4% articaine HCL with 1:200,000 epinephrine as compared with those of 4% articaine HCl with 1:100,000 epinephrine and 4% articaine HCl without epinephrine. As a result of the study, no difference was found between the two different epinephrine-containing formulas in terms of anesthesia efficiency, but the effectiveness of the formula without epinephrine was found to be lower action compared to the others. Lidocaine has a natural vasodilator effect. Studies have shown that epinephrine at different concentrations (1:100000 and 1:200000) added to lidocaine does not affect the anesthetic efficacy (30). In our study, however, since the anesthesia method which provides direct blockade of the nervus alveolaris inferior was utilized, the anesthetic agent was not required to penetrate cortical bone, and there was not any difference between the groups in terms of pain perception during the operation. For IANB, application as close to the nerve as possible and technically correct application are of primary importance. Bone density classification of Misch was used in our study using CBCT and measuring HU values. It is a classification that covers whether the bone is cortical or trabecular. Although there are studies that state that articaine has better cortical bone penetration, no relationship between bone structure and IANB anesthesia could be seen when implant surgery was performed.

## Conclusions

According to the results of the study, there is no difference between groups when VAS scale results are evaluated for 4% articaine and 2% lidocaine in terms of IANB which is utilized with the purpose of providing adequate anesthesia in posterior mandible implant surgeries.
